# Supplementary material for: Host response biomarkers of tuberculosis recurrence and treatment failure
Source: Commun Med (Lond). 2026 Feb 14;6:184. doi: 10.1038/s43856-026-01424-w (PMC13047042; doi:10.1038/s43856-026-01424-w)
Supplement: Supplementary file 2 — Supplementary Material [file 43856_2026_1424_MOESM2_ESM.pdf]

SUPPLEMENTARY MATERIALS

Table of Content

Supplementary Figure 1 2
Supplementary Table 1| 3
Supplementary Figure 2 4
Supplementary Table 2 5
Supplementary Figure 3 6
Comparison of Purified Protein Derivative (PPD) and MTB125 peptide pool for defining MTB-specific T cell frequencies and activation status 7
Supplementary Figure 4 8
Supplementary Figure 5 9
Supplementary References 10

Supplementary Figure 1| Gating strategy for *Mycobacterium tuberculosis* (MTB)-specific CD4 T cells.

Lymphocytes were first gated based on side scatter (SSC-A) versus forward scatter area (FSC-A), followed by selection of singlets. T cells were identified using the CD3 marker. CD4 T cells were then gated accordingly. IFNγ expression was used to identify *Mycobacterium tuberculosis*-specific CD4 T cells responding to MTB 125 stimulation. The frequency of MTB-specific IFNγ-positive CD4 T cells was subsequently assessed using CD38, CD27, HLA-DR, and Ki67 for downstream analyses.

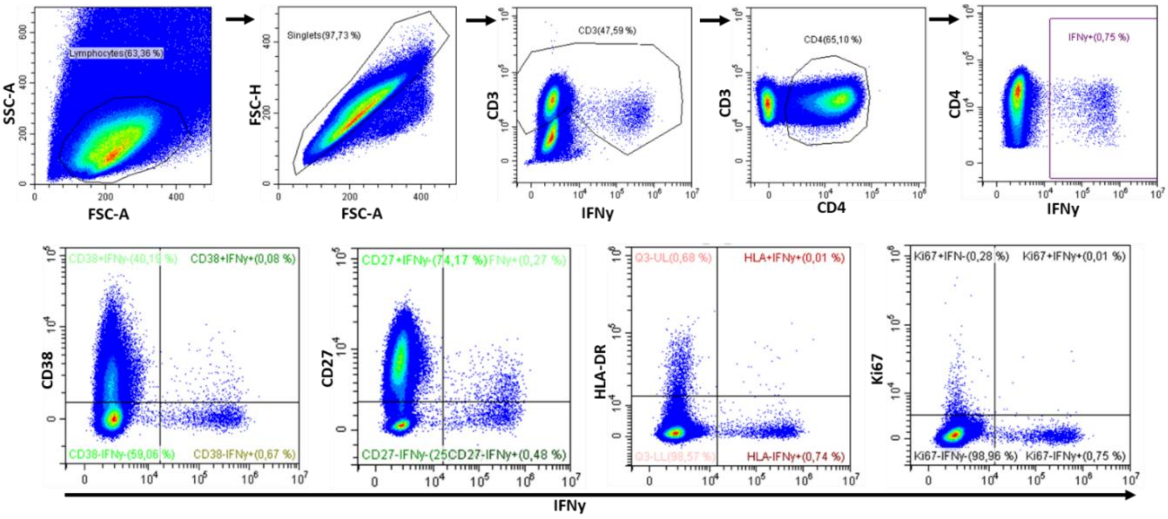

Supplementary Table 1| Transcriptomic Signatures Tested, Calculation Formulas, and Gene Identifier Mapping. Signatures tested are Sweeney 3 (1), RISK6 (2), and MAMS_6 (Ahmed et al., under review). Calculations use log-normalized gene expression, with gene names matched to Ensembl IDs as shown.

Signature	Calculation	Symbol to ID mapping
Sweeney 3	KLF2 – (GBP5 + DUSP3)/2	KLF2 : ENSG00000127528<br>GBP5 : ENSG00000154451<br>DUSP3 : ENSG00000108861
RISK 6	$\sqrt[3]{\mathrm{GBP2} * \mathrm{FCGR1B} * \mathrm{SERPING1}}$<br>$- \sqrt[3]{\mathrm{TUBGCP6} * \mathrm{TRMT2A} * \mathrm{SDR39U1}}$	GBP2 : ENSG00000162645<br>FCGR1B : FCGR1BP : ENSG00000198019<br>SERPING1 : ENSG00000149131<br>TUBGCP6 : ENSG00000128159<br>TRMT2A : ENSG00000099899<br>SDR39U1 : ENSG00000100445
MAMS 6	$\mathrm{mean}(\mathrm{AIM2}, \mathrm{FCGR1B}, \mathrm{CD274},$<br>$\mathrm{GBP1}, \mathrm{SMARCD3}, \mathrm{FLVCR2})$	AIM2 : ENSG00000163568<br>FCGR1B : FCGR1BP : ENSG00000198019<br>CD274 : ENSG00000120217<br>GBP1 : ENSG00000117228<br>SMARCD3 : ENSG00000082014<br>FLVCR2 :<br>ENSG00000119686

Supplementary Figure 2| Assessment of CD38+ MTB-specific CD4 T cell frequencies to differentiate stages of TB infection. Comparative analysis of CD38+ MTB-specific CD4 T cell frequencies (%IFNγ+) in individuals with TB disease (active TB (aTB) n=40 or incipient TB (iTB) n=13) compared to latent TB infection (n=29) (a). A color gradient represents the frequency of CD38+ MTB-specific T cells (green <24.5, yellow <31.6, orange <43.4, red >43.4). ROC analysis (b) identifies 31.6% as the optimal threshold to distinguish TB disease (aTB/iTB) from latent TB infection (sensitivity: 87%, CI: 75.2 %-93.5%; specificity: 90%, CI: 73.6%-96.4%). The area under the ROC curve (AUC) was 0.91 (95% CI: 0.84–0.99; $p < 0.0001$). Frequencies >43.4% identify TB disease (sensitivity >90%) and <24.5% identify latent TB infection (specificity >90%). Data are based on two African studies, TB-MAMS and Africos *(3, 4)*. In this Figure, we used the terms aTB, iTB, and latent TB to align with the original manuscript.

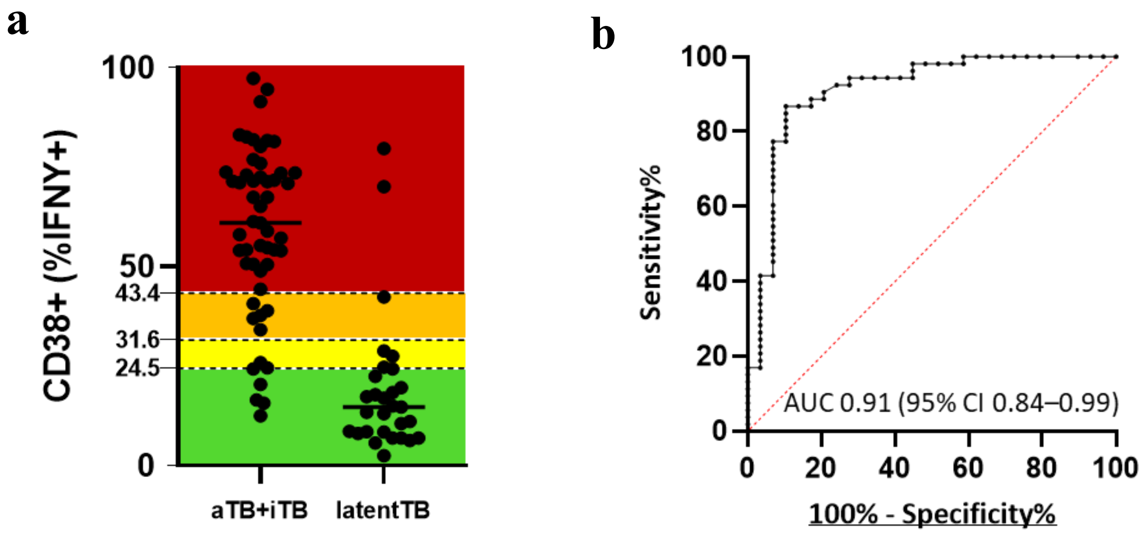

Supplementary Table 2| Presentation of demographic and clinical characteristics of the tuberculosis treatment failure and recurrence subgroups: non-converter, reverter at EOT and recurrence after EOT compared to a successfully treated control group. Data was 85% complete for all variables except CRP, which was available for 67% of participants in both groups. 'NA' indicates variables with ≥50% missing values. Abbreviations: EOT= end of treatment, Mzb=Mozambique, Tz=Tanzania, Gmb=Gambia, Rsa=Republic South Africa, BL=Baseline, BMI=body mass index.

	Treatment failure and recurrence group			Control group
	Non-converter n = 7	Reverter at EOT n = 14	Recurrence after EOT n = 19	n = 37
Matching Criteria				
Site Mzb/Tz/Gmb/Rsa, %	14/43/43/0	7/43/21/29	0/47/21/32	8/46/24/22
Sex (male), %	100	57	63	65
HIV pos. at BL, %	14	21	53	38
Age, median (IQR)	28.0 (24.0 – 36.0)	36.5 (31.2 – 44.5)	36.0 (32.0 – 43.0)	33.0 (30.0 – 42.0)
BMI, median (IQR)				
Baseline	16.7 (15.2 – 17.3) *	18.3 (17.0 – 19.0)	18.4 (17.0 – 19.9)	18.9 (17.5 – 21.4)
Month 6	17.1 (16.5 – 18.1) ***	20.0 (18.1 – 21.1)	20.8 (19.5 – 21.6)	21.0 (19.7 – 22.7)
CRP Value Baseline, median (IQR)	NA	78.2 (59.5 – 94.6) **	104.8 (66.9 – 146.7)	161.0 (81.4 – 243.4)
Microbiological results				
Sputum smear positivity, n (%)				
Screening/Baseline	6 (86)	14 (100)	19 (100)	26 (81)
Month 2	4 (67) ***	0 (0)	1 (6)	1 (3)
Month 6	5 (83) ***	1 (7)	1 (6)	0 (0)
Liquid culture positivity, n (%)				
Screening/Baseline	7 (100)	14 (100)	19 (100)	34 (100)
Month 2	6 (86) ***	5 (46)	9 (56) **	5 (16)
Month 6	6 (86) ***	10 (71) ***	0 (0)	0 (0)
Solid culture positivity, n (%)				
Screening/Baseline	5 (83)	13 (93)	15 (79)	28 (78)
Month 2	3 (43)	2 (15)	6 (38) *	3 (9)
Month 6	4 (43) ***	2 (15)	0 (0)	0 (0)
Chest X Ray – Ralph Score, median (IQR)				
Month 6	30.0 (13.0 – 65.0) *	5.0 (2.8 – 10.0)	15.0 (5.0 – 50.0)	5.0 (5.0 – 28.8)
Number of symptoms (Cough, bloody cough, fever, weight loss, night sweats, body weakness)				
BL, ≥2/1/0 in %	100/0/0	100/0/0	100/0/0 *	87/13/0
M6, ≥2/1/0 in %	29/43/29	14/29/57	6/33/61	14/32/54

CRP: C-reactive protein, an unspecific inflammation marker.
Ralph Score: Radiographic score which considers the extent of lung infiltrate and the presence or absence of cavities.
** Percentages are based on valid results only; indeterminate samples (≤21% per timepoint) were excluded from the analysis.*
*Significances codes (p-value): * p ≤ 0.05, ** p ≤0.01, *** p ≤ 0.001.*
Comparison was always made with the control group. Statistical tests used: Wilcoxon rank sum test, Wilcoxon rank sum exact test, and Fisher's exact test, all tests were performed two-sided.

Supplementary Figure 3| Frequency and phenotypic profiles for MTB-specific CD4 T cells stimulated with MTB 125 (125 frequently recognized *Mycobacterial tuberculosis* peptides) or PPD (Purified Protein Derivative) (x-axis) across all participants (control group and TB treatment failure and recurrence group) and timepoints (BL – M12). The y-axis in figure a shows the frequency of IFNγ+ events (five PPD values >1.5% not plotted). The expression of the activation markers CD38 (b), HLA-DR (c), Ki 67 (d) and the maturation marker CD27 (e) is shown for all visit times together. In figure F, the expression of the CD27 marker (x-axis) is shown on the y-axis over time. The box represents the interquartile range (IQR), with whiskers extending to the farthest points within 1.5 times the IQR. Statistical analysis was done using the Wilcoxon signed-rank Test. Significances codes (p-value): * $p \leq 0.05$, ** $p \leq 0.01$, *** $p \leq 0.001$.

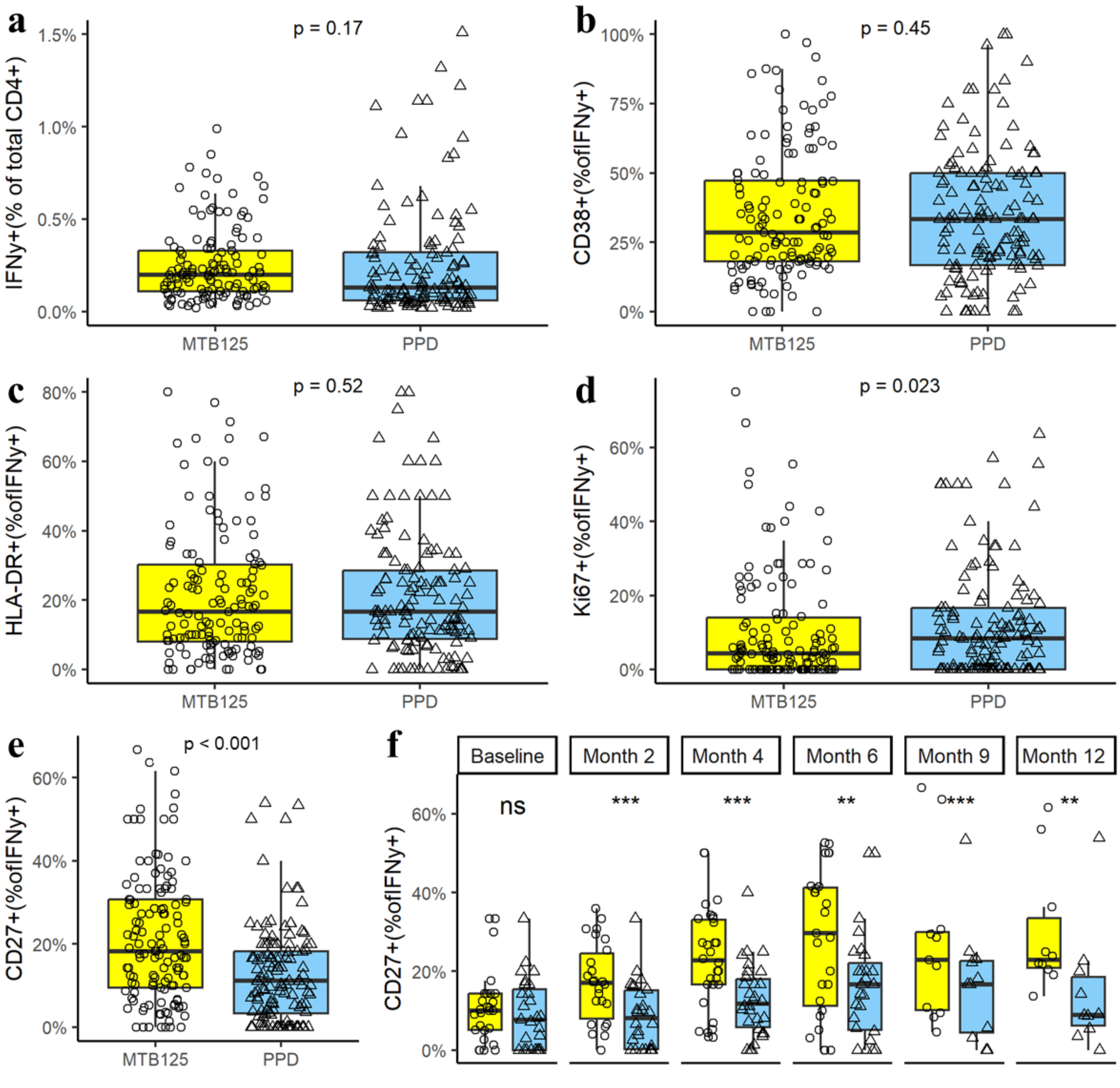

Comparison of Purified Protein Derivative (PPD) and MTB125 peptide pool for defining MTB-specific T cell frequencies and activation status

PBMC samples (n=190) were stimulated with a previously described peptide pool of 125 known *Mycobacterium tuberculosis* peptides (MTB125) (5, 6) and PPD. Exclusion rates due to poor quality were similar for both MTB125 (16%, n=28) and PPD (16%, n=31; data not shown). No significant difference in the percentage of MTB-specific CD4 T cells was observed between stimulation with MTB125 and PPD, when comparing all available paired samples (n=131) across all visits (supplementary figure 3a).

We then investigated whether using these two MTB antigens influences the expression of activation (CD38, HLA-DR, Ki67) and maturation markers (CD27) on MTB-specific CD4 T cells (supplementary figure 3 b-e). Frequencies of CD38 and HLA-DR expressing MTB-specific CD4 T cells were similar with PPD or MTB125 (supplementary figure 3b and 2c). However, the frequency of Ki67+ MTB-specific CD4 T cells were 1.9-fold higher with PPD stimulation ($p=0.023$, supplementary figure 3d). The most significant difference was in CD27 expression, which was higher after MTB125 stimulation (median = 18.2%) compared to PPD (median = 11.1%, $p<0.001$, supplementary figure 3e). From M2 to M12 after TB treatment initiation, a higher proportion of MTB-specific CD4 T cells express the CD27 marker in MTB125-stimulated samples compared to PPD-stimulated ones ($p<0.05$ for all treatment time points, Figure 2f). These results confirm that MTB125 is as effective as PPD in detecting MTB-specific T cells and assessing their activation profile throughout TB treatment, demonstrating that the synthetic MTB125 pool can replace PPD for evaluating MTB-specific T-cell activation. Therefore, MTB125 results were used for further analysis.

Supplementary Figure 4| Comparative analysis of MTB-specific CD4 T cell activation and maturation marker expression in individuals before TB reversion/recurrence and in successfully treated controls. (a) Frequency of MTB-specific CD4 T cells expressing the activation marker CD38 before reversion or recurrence, compared to the successfully treated control group. The last available blood sample from each participant before reversion or recurrence was categorized into two groups: M4&M6 and M9&M12. (b) shows the time intervals between the last PBMC sample and recurrence diagnosis for the M9&M12 group. The green dashed line marks the end of TB treatment. (c) provides an overview of the composition of the M4&M6 and M9&M12 reverter and recurrence subgroups. (d) Frequency of MTB-specific CD4 T cells expressing the maturation marker CD27 before reversion or recurrence at M4&M6, compared to the successfully treated control group. Statistical analysis was done using the Wilcoxon Rank Sum Test.

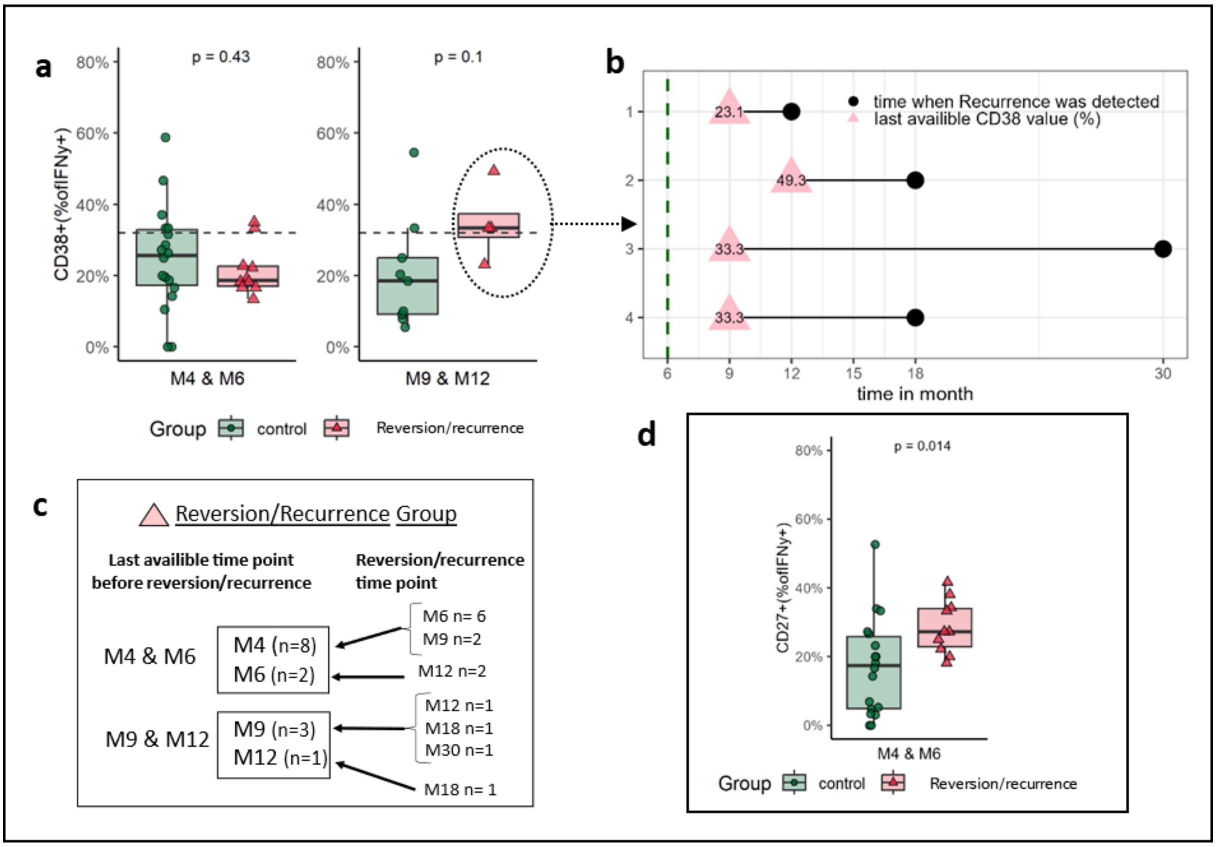

Supplementary Figure 5| Representative flow cytometry dot plots for phenotypic characterization of MTB-specific CD4 T cells in three groups: successfully treated tuberculosis ("Control") (a), tuberculosis recurrence at M18 (b), and an active tuberculosis case with persistent microbiological positivity throughout treatment ("Non-converter") (c). Unstimulated negative controls (NC) are shown alongside MTB-stimulated cells (MTB+). Dynamic changes in CD38 expression within each group are shown at baseline, month 2, month 4, and month 6 for panels a and c, and at months 2, 6, 9, and 12 for panel b.

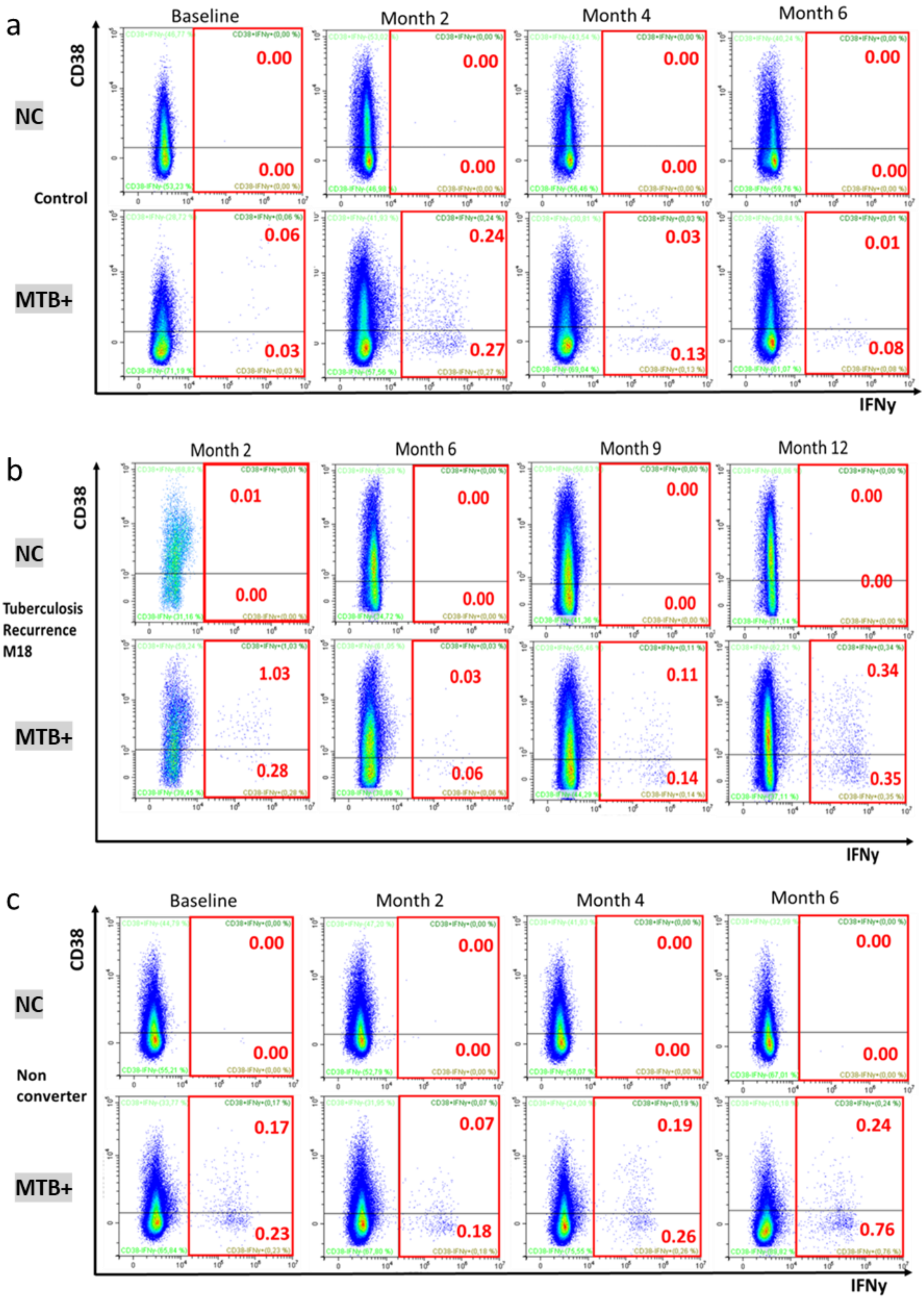

Supplementary References

1. Sweeney TE, Braviak L, Tato CM, Khatri P. Genome-wide expression for diagnosis of pulmonary tuberculosis: a multicohort analysis. Lancet Respir Med 2016; 4(3):213–24.

2. Penn-Nicholson A, Mbandi SK, Thompson E, Mendelsohn SC, Suliman S, Chegou NN et al. RISK6, a 6-gene transcriptomic signature of TB disease risk, diagnosis and treatment response. Sci Rep 2020; 10(1):8629.

3. Kroidl I, Ahmed MIM, Horn S, Polyak C, Esber A, Parikh A et al. Assessment of tuberculosis disease activity in people infected with Mycobacterium tuberculosis and living with HIV: A longitudinal cohort study. EClinicalMedicine 2022; 49:101470.

4. Ahmed MIM, Ntinginya NE, Kibiki G, Mtafya BA, Semvua H, Mpagama S et al. Phenotypic Changes on Mycobacterium Tuberculosis-Specific CD4 T Cells as Surrogate Markers for Tuberculosis Treatment Efficacy. Front Immunol 2018; 9:2247.

5. Lindestam Arlehamn CS, McKinney DM, Carpenter C, Paul S, Rozot V, Makgotlho E et al. A Quantitative Analysis of Complexity of Human Pathogen-Specific CD4 T Cell Responses in Healthy M. tuberculosis Infected South Africans. PLoS Pathog 2016; 12(7):e1005760.

6. Riou C, Du Bruyn E, Ruzive S, Goliath RT, Lindestam Arlehamn CS, Sette A et al. Disease extent and anti-tubercular treatment response correlates with Mycobacterium tuberculosis-specific CD4 T-cell phenotype regardless of HIV-1 status. Clin Transl Immunology; 2020. (vol 9).
