# Supplementary material for: Host response biomarkers of tuberculosis recurrence and treatment failure
Source: Commun Med (Lond). 2026 Feb 14;6:184. doi: 10.1038/s43856-026-01424-w (PMC13047042; doi:10.1038/s43856-026-01424-w)
Supplement: Supplementary file 3 — Description of Additional Supplementary files [file 43856_2026_1424_MOESM3_ESM.pdf]

Description of Additional Supplementary Files

File name: Supplementary Data 1

Description: Source data underlying the main figures, including the TAM-TB cohort and the relevant clinical datasets
